# Case Report: Molecular Features and Treatment Options for Small Bowel Adenocarcinoma

**DOI:** 10.3389/fonc.2021.593561

**Published:** 2021-03-10

**Authors:** Miguel Cordova-Delgado, Gonzalo Pizarro, Mauricio P. Pinto, Maria Elisa Herrera, Marcelo Garrido

**Affiliations:** ^1^Hematology and Oncology Department, Faculty of Medicine, Pontificia Universidad Católica de Chile (PUC), Santiago, Chile; ^2^Faculty of Chemical and Pharmaceutical Sciences, Universidad de Chile, Santiago, Chile; ^3^Medical Oncology Department, Hospital de Valdivia, Valdivia, Chile; ^4^Medical Oncology Department, Clínica Alemana de Valdivia, Valdivia, Chile

**Keywords:** case report, small bowel adenocarcinoma, comprehensive genomic profiling, precision oncology, therapeutic options

## Abstract

Small bowel adenocarcinoma (SBA) is a rare malignancy characterized by poor prognosis. Recent efforts have sought to elucidate the genetic landscape and the molecular drivers behind this disease. Herein, we report the main molecular alterations in two metastatic (stage IV) SBA patients. Interestingly, one of them had gene alterations that affected signaling pathways previously described for SBA. However, a second patient displayed previously unreported alterations in this particular tumor type. Based on these findings we discuss potential treatment options for patients affected by this rare, aggressive disease.

## Introduction

Small bowel adenocarcinoma (SBA) is a rare disease representing <3% of all gastrointestinal cancers (1). Given its asymptomatic presentation and the lack of effective diagnostic tools, most cases are diagnosed at advanced stages (2), partially explaining its poor prognosis. Typically, median survival for untreated stage IV patients is <6 months (3, 4). Risk factors for SBA include the Lynch syndrome, familial adenomatous polyposis, celiac disease, Crohn's disease and the Peutz-Jeghers syndrome (5, 6).

Palliative chemotherapy is the preferred standard treatment for advanced stage unresectable SBA. Unfortunately, given its low prevalence only a few treatment protocols have been reviewed throughout the literature. Given its anatomical proximity, current chemotherapy protocols for SBA are based on regimens used for colon cancer including mFOLFOX6 and FOLFIRI (7). Studies have demonstrated a median overall survival of 12–18 months (8–10) using fluoropyrimidines/platinum compound combinations. Thus, in the absence of a gold standard these are usually the recommended approach (7).

**Figure 1:**
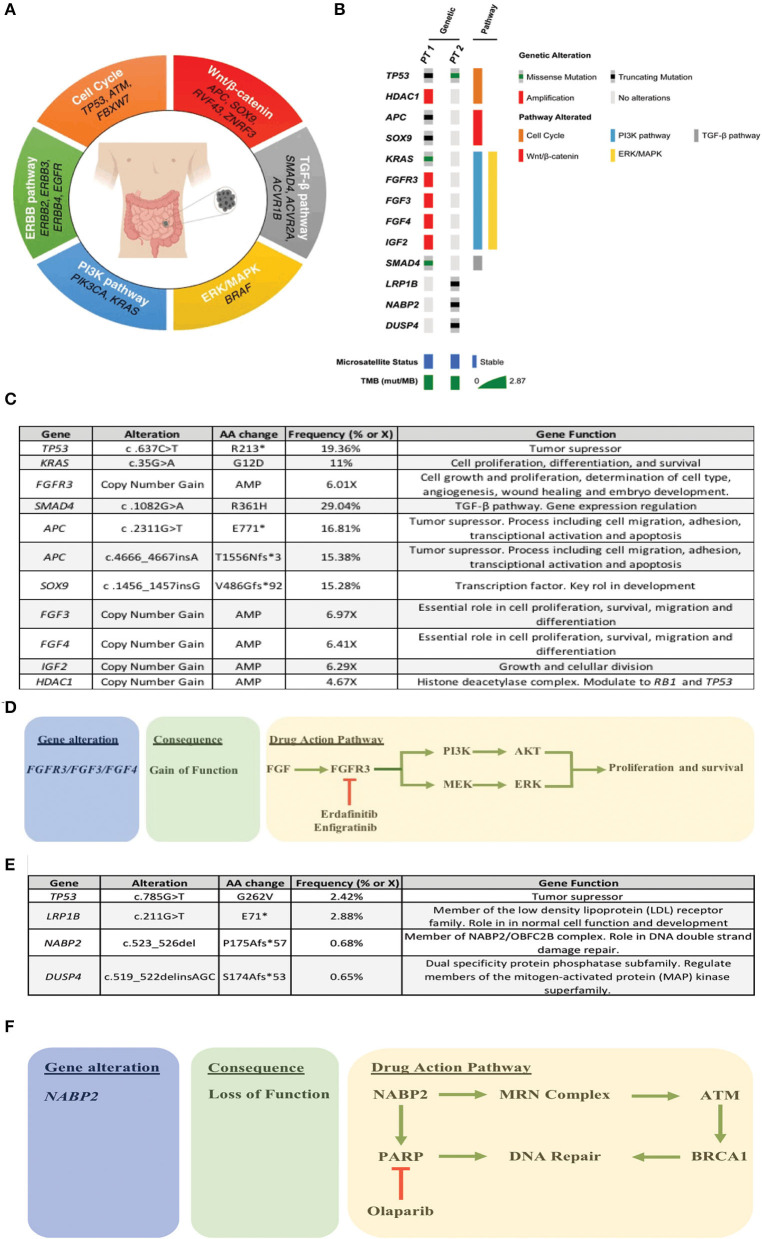
**(A)** Recurrently altered molecular pathways in SBA. **(B)** Oncoprint of the two patients, where the most relevant alterations/pathways, microsatellite status, and TMBx were included. **(C)** Summary of patient 1 alterations. **(D)** Treatment rationale based on molecular alterations in patient 1. **(E)** Summary of patient 2 alterations. **(F)** Treatment rationale based on molecular alterations in patient 2.

As pointed above, clinical SBA treatments mimic the strategies used for colorectal cancer (CRC) patients. However, there are evident and substantial differences between these two tumor types, in terms of incidence and mortality (11). Schrock et al. (12) reported relevant molecular differences among SBA, CRC and gastric cancers; SBAs are characterized by microsatellite instability and a high tumor mutational burden whereas up to 80% of CRCs harbor APC mutations. In contrast, APC mutation rate in SBA ranges between 7 and 13%. Regarding targeted therapies, a study demonstrated that anti-EGFR therapies were ineffective for metastatic SBA patients (13) compared to CRC. In this study, Gulhati et al. selected RAS wild-type patients, however panitumumab did not yield any response by RECIST. There are several anti-EGFR resistance mechanism pathways and some of these are overexpressed in SBA. One example is HER2-overexpression, also alterations in genes encoding key EGFR-dependent intracellular signaling transducers, such as KRAS, NRAS, BRAF, PIK3CA, MEK, or ERK. Despite this, a recent retrospective analysis of 13 metastatic SBA patients concluded that the combination of anti-EGFR inhibitors and chemotherapy could be potentially beneficial for these patients (14). Therefore, the evidence in this topic is still inconsistent. Consequently, the elucidation of SBA molecular drivers becomes crucial to develop more personalized therapies and improve patient survival. Recent efforts have identified the main pathways altered in SBA these are summarized in Figure 1A; these include Wnt/β-catenin, *ERBB*, ERK/MAPK, PIK3, cell cycle, and TGF-β signaling pathways. As occurs in several cancer types, *TP53* is the most frequently mutated gene, suggesting a potential tumorigenic role (15, 16). Another frequently reported pathway is Wnt/β-catenin, this is usually involved in cell growth, proliferation and differentiation, suggesting a critical role in SBA tumorigenesis and poor prognosis (17).

Herein, we report the main molecular features of two metastatic (stage IV) SBA patients. Interestingly, signaling pathway related gene alterations in these patients were notoriously different. First, a patient that displayed previously reported SBA alterations and a second patient with a completely different set of previously unreported alterations for SBA. Based on these results, we discuss potential therapeutic options.

## Presentation of Cases

### Patient 1

Patient was a male, 51-year-old with no history of chronic diseases or relevant surgeries. Patient refers iron-deficiency anemia and weight loss over the last 7 months. Abdominal ultrasound revealed liver lesions suggesting metastatic disease. A subsequent upper gastrointestinal endoscopy (UGE) found a non-ulcerated tumor located in the third portion of the duodenum. Biopsy found liver infiltration by a tubular moderately differentiated adenocarcinoma. PET/CT confirmed a primary tumor in the duodenum along with bilobar, pulmonary, liver, and lymph node metastases. At diagnosis, patient had a CEA = 3.2 ng/ml, mild anemia, and normal liver and kidney function.

Liver metastasis tissue (FFPE) was analyzed using a panel of 688 cancer-related genes (Sentis Cancer + Discovery, BGI) that found 17 somatic mutations, 11 of those were clinically relevant (Figure 1C) and six were variants with uncertain significance (VUS). No pathogenic germline mutations were detected in a total of 63 susceptibility genes evaluated. The analysis also reported 2.87 mutations per MB (mut/MB), microsatellite stability (MSS) and a number of relevant mutations (Figure 1B) including two non-sense *APC* mutations and a non-sense *SOX9* mutation, both associated with the WNT signaling pathway. Other alterations were gain-of-function gene amplifications in *FGFR3, FGF3, FGF4*, and *IGF2*; these are all related to the PI3K signaling pathway. Also, a missense *SMAD4* mutation. Following analysis, patient started palliative FOLFOX, obtaining a partial response after 6 cycles. After completion of 12 cycles, patient was then switched to capecitabine for maintenance, but returned to the clinic referring pain in the right hypochondrium and in the lumbar region after a month. A PET/CT scan indicated progression of liver and lung metastases, and novel L5 and left iliac bone lesions, further confirmed by MRI. Patient then was scheduled for palliative radiotherapy against bone metastases. Unfortunately, given its rapid deterioration patient did not receive matched therapy according to FGFR alterations. Timeline of relevant therapies and clinical follow up are presented in Table 1.

**Table 1:** Timeline of relevant therapies and clinical follow up of patient 1.

**Time period**	**Therapies**
June 2019	Patient presented with liver, lung and bone metastases
July/Nov. 2019	mFOLFOX6 (oxaliplatin 85 mg/m^2^, leucovorine 400 mg/m^2^, 5-fluorouracil 400 mg/m^2^ as a bolus and 2,400 mg/m^2^ as a 46-h infusion) every 2 weeks per 12 cycles
Jan./Feb. 2020	Capecitabine 1,000 mg/m^2^ twice a day
Feb. 2020	Progressive disease, with new liver, lung and bone metastases. Palliative radiotherapy to liver and bone metastases (8 Gy 1 fraction)

### Patient 2

Patient was a 41-year-old female with no history of chronic disease or relevant surgeries. Within the last 4 months patient refers occasional abdominal pain and vomiting. An UGE and a colonoscopy revealed no pathological findings. An abdominal/pelvic CT found a solid mass in the right ovary and edema in the proximal jejunal wall. Patient plasma CA-125, CA19-9, and CEA levels were elevated. An exploratory laparotomy found several implants in the omentum and a right ovarian mass that were surgically removed. Further exploration found an annular and stenosing tumor in the proximal jejunum that was resected by entero-anastomosis and peritoneal carcinomatosis. A biopsy of the jejunal tumor showed a poorly differentiated mucinous adenocarcinoma with signet ring cells infiltrating the visceral peritoneum. In addition, a signet ring cell mucinous adenocarcinoma suggesting a Krukenberg tumor was observed in the right ovary. Afterwards, patient started first-line mFOLFOX6 obtaining a partial response. Unfortunately, treatment was suspended at cycle 9 due to toxicity. Clinical follow up continued until peritoneal progression 6 months later, restarting mFOLFOX6 plus Bevacizumab. Three months later an abdominal/pelvic CT revealed further peritoneal progression.

As described above, ovarian (left) metastatic tissue (FFPE) was analyzed by a panel of 688 cancer-related genes (Sentis Cancer + Discovery, BGI) that found 15 somatic mutations, four were clinically relevant (Figure 1E) and 11 VUS. Similar to patient 1 no germinal pathogenic mutation were found. Patient was classified as MSS and had a tumor mutational burden (TMB) of 2.51 mut/MB (Figure 1B). Most relevant alterations included a *TP53* missense mutation, a *LRP1B* non-sense mutation, and frameshift mutations on *NABP2* and *DUSP4*. Unlike *TP53*, gene alterations in *LRP1B, NABP2*, and *DUSP4* have not been previously reported in SBA. In particular, *LRP1B* is a tumor suppressor gene and member of the LDL receptor family. *NABP2* is a DNA repair gene, particularly for double-strand breaks and also related to *ATM* activity. Finally, *DUSP4* codifies for a MAPK, ERK, SAPK/JNK, p38 phosphatase that downregulates their activity. In view of these findings, patient started Olaparib (300 mg/12 h PO) in February 2020. Unfortunately, patient reported anorexia, nausea and diarrhea (all G2) after the first cycle. Then, dose was reduced to 200 mg/12 h, obtaining a stable disease. However, in May 2020 patient displayed a malignant intestinal obstruction that required surgery. Subsequently, patient evolved with good oral tolerance and without new episodes of obstruction and started systemic FOLFIRI. To date, patient maintains a stable disease. Timeline of relevant therapies and clinical follow up are presented in Table 2.

**Table 2:** Timeline of relevant therapies and clinical follow up of patient 2.

**Time period**	**Therapies**
March 2018	Patient presented with right ovarian tumor. Laparotomy: Adnexectomy plus omentectomy, jejunal tumor plus peritoneal carcinomatosis was observed. Jejunal resection
Apr./Aug. 2018	mFOLFOX6 (oxaliplatin 85 mg/m^2^, leucovorin 400 mg/m^2^, 5-fluoruracil 400 mg/m^2^ as a bolus and 2,400 mg/m^2^ as a 46-h infusion) every 2 weeks per nine cycles
Apr./Nov. 2019	Peritoneal progression. mFOLFOX6 (oxaliplatin 85 mg/m^2^, leucovorin 400 mg/m^2^, 5-fluoruracil 400 mg/m^2^ as a bolus and 2,400 mg/m^2^ as a 46-h infusion) plus Bevacizumab 5 mg/kg every 2 weeks
Dec. 2019	Second peritoneal progression
Feb. 2020	Olaparib 300 mg/12 h PO. After cycle #1 patient reported anorexia, nausea and diarrhea (all G2) after the first cycle. Then, dose was reduced to 200 mg/12 h
Feb./May 2020	Stable disease
May 2020	Malignant intestinal obstruction
May/Dec. 2020	Systemic treatment with FOLFIRI (irinotecan 180 mg/m^2^, day 1; leucovorin 400 mg/m^2^ intravenously, day 1; 5-fluorouracil 400 mg/m^2^ intravenous bolus, day 1; and 2400 mg/m^2^ in 22 hrs intravenously, continuous infusion days 1 and 2) every two weeks, maintaining stable disease

## Discussion

Advanced stage SBA is characterized by poor survival/prognosis and low response rates to systemic treatments. Recently, a study reported a 12.7-month median OS for stage IV SBA (95% CI, 10.7–16.4) in a prospective cohort of 347 patients. Moreover, 85.2% (101/123) of unresectable patients in this study were treated with fluoropyrimidine/platinum-based chemotherapy regimens (18), reflecting the lack of molecularly targeted therapies for patients in this setting. Consequently, molecular characterization studies could uncover clinically relevant, actionable alterations aiming to improve SBA patient prognosis.

### Therapeutic Options Based on Molecular Findings in Patients With Advanced SBA

Our analysis found that patient 1 was microsatellite stable (MSS) and had a low TMB. These are commonly associated to a poor response to Immune Checkpoint Inhibitor (ICI). In addition, patient displayed a series of gain-of-function *FGFR* amplifications. These are associated to increased tumor cell proliferation and survival *via* the PI3K/ERK pathway. In this context, a variety of multi-targeted tyrosine kinase inhibitors could be used as therapeutic options, including selective and non-selective FGFR tyrosine kinase inhibitors such as dovitinib, ponatinib, pazopanib, nintedanib, lucitanib, brivanib, lenvatinib, erdafitinib (JNJ 42756493), infigratinib (BGJ398), and Rogaritinib (BAY 1163877). As shown in Figure 1D, one of the reported cases had a FGFR3 gain-of-function amplification. Interestingly, both erdafitinib and infigratinib have demonstrated a promising anti-tumor activity in patients with altered FGFR3. Specifically, erdafitinib binds FGFR2 and FGFR3 by inhibiting FGF activity, eventually leading to cell death. A phase II trial demonstrated an ORR = 49% in FGFR3 mutants vs. a 16% in patients with FGFR2/3 fusions (19). This study led to the approval of erdafitinib for second-line advanced urothelial cancer patients with FGFR2/3 alterations (20). A subsequent study estimated the off-label use of several drugs, including erdafitinib and determined that the potential off-label erdafitinib users could be up to 3 times greater than under the current recommendations (21). On the other hand, infigratinib is a potent FGFR1-3 inhibitor with demonstrated single-agent antitumor activity in FGFR3 mutant early-stage urothelial cancer patients (22). A phase II trial that seeks to evaluate infigratinib in advanced/metastatic solid tumors with FGFR abnormalities is currently enrolling patients (ClinicalTrials.gov = IDNCT04233567). This suggests that targeting the FGFR signaling pathway might be a therapeutic option for a subset of SBA patients with a molecular profile.

As occurs with patient 1, our patient 2 was unlikely to respond to ICI treatments, due to her low TMB and her microsatellite stability (MSS) status. In fact, patient's mutation profile does not display any potential markers for targeted therapy. However, based on the observed alterations we could speculate on an alternative approach. Normally, *NABP2* participates in the hSSB1 complex (NABP2/OBFC2B). Among other functions this complex repairs double stranded damaged DNA. *In-vitro* studies have reported that NABP2/OBFC2B loss is associated to *ATM* deficiency. In turn, a reduced ATM function correlates with PARP-inhibitor sensitivity (23, 24). Therefore, *NAPB2* mutation on this patient could be associated with a favorable response to PARP inhibitors, such as olaparib (Figure 1F). Within this context, only a couple of studies have assessed the effect of PARP inhibitors in SBA patients. First, a case report of a *BRCA1* mutant small intestine mixed adeno-neuroendocrine carcinoma patient showed susceptibility to olaparib in combination with carboplatin/paclitaxel (25). Second, studies report a relatively high proportion of *BRCA1/2* mutants among SBA patients that range from 7 to 36% (15, 26). Given the demonstrated efficacy of PARP inhibitors (27) in germline/somatic *BRCA1/2* mutants these findings warrant further clinical investigation in this rare cancer.

In addition to BRCA, other potentially actionable alterations have been recently described for SBA, opening the possibility of a more personalized treatment, especially for advanced stage patients. For example, studies recurrently report *ERBB2* mutations/amplifications which opens the gate for anti-ERBB2/anti-HER2 therapies such as trastuzumab in specific subsets of patients (26, 28, 29). Similarly, studies have shown 14% (15) and 18% (26) of microsatellite instability (MSI) among SBA patients. Interestingly, Aparicio et al. used molecular and immunohistochemical analyses of Mismatch Repair (MMR) proteins and reported a 23% of MMR deficiency in SBA tumors (*n* = 61) they also observed that MMR deficiency was associated with non-metastatic patients (30). In addition, a study by Giuffrida et al., reported a 26% of PD-L1+ by combined positive scores (CPS) in a cohort of 121 SBA patients (31). A recent study assessed the anti-tumor efficacy of Pembrolizumab (anti-PD-1) in MSI-high or dMMR non-CRC patients and found that small intestine cancer patients (n=19) had an impressive ORR of 42.1% and a median PFS of 9.2 months (32). Hence, given the promising results in MSI or dMMR SBA a subset of these patients might actually benefit from anti-PD-1 therapies. However, there is no evidence demonstrating that high expression of PD-L1 could serve as a marker of anti-PD1 response.

On the other hand, surgery could be an option for SBA patients. The ARCAD-NADEGE study (33) evaluated the role of curative intent resection of metastases in adenocarcinoma of the small intestine. This study found that ~90% of metastatic patients underwent surgical resection and received oxaliplatin-based chemotherapy (either perioperative or adjuvant). Metastatic sites included peritoneum (29.4%), liver (26.5%), lymph nodes (11.8%), lung (2.9%), multiple (14.7%), and other (14.7%). Median OS for these patients was 28.6 months, and in about 40% of cases this value was >36 months. Furthermore, this study found that the degree of differentiation, negative resection margins and the use of adjuvant chemotherapy with oxaliplatin were associated with better OS. Although this was based on a limited number of cases, these results exceed those published in historical series that included patients treated with chemotherapy alone. Therefore, surgical resection of metastases could be an option for a subset of SBA patients that have the possibility to achieve negative (R0) margins.

**Figure 2:**
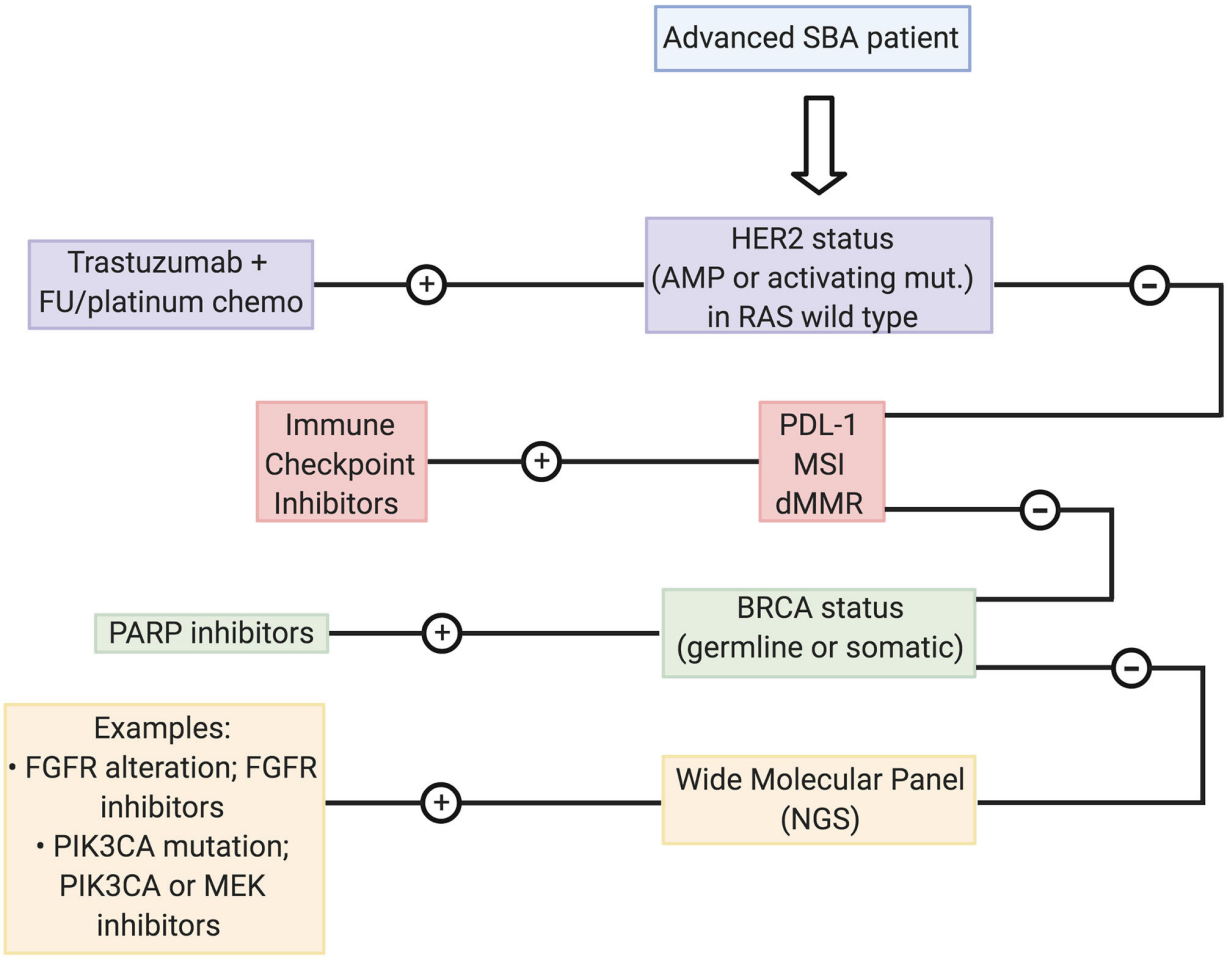
Management plan proposed in advanced SBA including treatment based on molecular findings.

As explained above, due to its aggressiveness and the lack of clinical efficacy of targeted treatments therapeutic options for advanced SBA patients are largely limited to palliative chemotherapy regimens. Based our data, we propose a management strategy for recurrent/metastatic SBA patients (shown in Figure 2). Briefly, we propose testing patients by a series of biomarkers associated with “actionable targets” previously reported for this malignancy. First step includes evaluation of *ERBB2/*HER2 status by IHC or DNA sequencing to determine overexpression or gain-of-function activating mutations, and an assessment of RAS status. Reports suggest that alterations in this pathway (KRAS/ PIK3CA/ BRAF) might cause anti-HER2 resistance (34). Next, an assessment of ICI response markers including PD-L1, MSI, or dMMR status by IHC, DNA sequencing, or PCR. Then a determination of *BRCA1/2* status that can be assayed by PCR or DNA sequencing. Finally, patients categorized as negative for all abovementioned biomarkers should undergo a more comprehensive analysis by an NGS panel searching for specific actionable alterations that would allow a more personalized treatment.

## Conclusion

Like most malignancies, SBAs are highly heterogeneous. Here we report 2 metastatic cases with distinctive molecular characteristics, revealing driver alterations that open the possibility of personalized (targeted) treatments. We propose a management plan based on “targeted therapy” markers. Our management plan should be further confirmed and validated by clinical trials, assessing the efficacy of these therapies.

## Data Availability Statement

The original contributions presented in the study are included in the article/supplementary material, further inquiries can be directed to the corresponding author/s.

## Ethics Statement

Ethical review and approval was not required for the study on human participants in accordance with the local legislation and institutional requirements. The patients/participants provided their written informed consent to participate in this study. Written informed consent was obtained from the individual(s) for the publication of any potentially identifiable images or data included in this article.

## Author Contributions

MC-D, GP, MP, MH, and MG: conceptualization, writing—original draft preparation, and formal analysis. GP, MH, and MG: data patients acquisition. All authors have read and agreed to the published version of the manuscript.

## Conflict of Interest

MG is an advisor for Merck Sharp & Dohme and Novartis, participates at the speakers' bureau for Bristol-Myers & Squibb and Bayer. Also receives research funding from Bristol-Myers & Squibb (Inst) and Novartis (Inst). Also, has received travel accommodations and expenses from Roche. The remaining authors declare that the research was conducted in the absence of any commercial or financial relationships that could be construed as a potential conflict of interest.
